# Prognostic Significance of DNA Repair Gene mRNA Expression in Early-Stage Breast Cancer: Insights into Clinical Relevance

**DOI:** 10.32604/or.2025.072222

**Published:** 2026-02-24

**Authors:** Ina Shehaj, Slavomir Krajnak, Katrin Almstedt, Yaman Degirmenci, Roxana Schwab, Kathrin Stewen, Walburgis Brenner, Annette Hasenburg, Marcus Schmidt, Anne-Sophie Heimes

**Affiliations:** 1 Department of Obstetrics and Gynecology, University Medical Center, Johannes Gutenberg-University Mainz, Mainz, 55131, Germany; 2 Department of Gynaecology and Obstetrics, Johann Wolfgang Goethe University, Frankfurt am Main, 60596, Germany

**Keywords:** *Ataxia-telangiectasia mutated (ATM)*, *Bloom helicase gene (BLM)*, *WRN RecQ Like Helicase (WRN)*, breast cancer (BC), gene expression analyses, survival

## Abstract

The prognostic and therapeutic roles of biological markers in early-stage breast cancer (eBC) warrant further investigation. Non-Breast Cancer (BRCA) genes, along with moderate- and low-penetrance breast cancer risk variant genes, are crucial for maintaining genome stability, yet their prognostic significance in eBC remains unclear. This study aimed to evaluate the impact of non-BRCA genes on clinical outcomes in eBC patients. Significant correlations were observed between the messenger ribonucleic acid (mRNA) expression levels of the genes *Ataxia-telangiectasia mutated (ATM), Bloom helicase gene (BLM)*, and *WRN RecQ Like Helicase (WRN)* and patient prognosis. High mRNA expression of ATM was associated with longer metastasis-free survival (MFS). Conversely, lower mRNA expression of BLM correlated with favorable outcomes, particularly in triple-negative tumors. Additionally, high levels of WRN mRNA expression were linked to significantly longer MFS compared to low expression levels. This study highlights the prognostic significance of ATM, BLM, and WRN in predicting survival outcomes in eBC patients.

**Background:**

The prognostic significance of various biological and non-BRCA genetic in early-stage breast cancer (eBC) remains unclear and warrants further investigation. This study therefore aimed to evaluate the prognostic impact of these genes on clinical outcomes in breast cancer.

**Methods:**

Patients included in this study were subdivided into two groups based on low and high messenger ribonucleic acid (mRNA) expression levels. Statistical analysis, including Kaplan-Meier curves, univariable, and multivariable Cox regression analyses, was performed to assess metastasis-free survival (MFS) of mRNA expression of non-BRCA genes. Subgroup analyses were also conducted among four different molecular subtypes of eBC.

**Results:**

Our analysis revealed significant correlations between mRNA-expression levels of *Ataxia-telangiectasia mutated (ATM), Bloom helicase gene (BLM)*, and *WRN RecQ Like Helicase (WRN)* and patient prognosis. High mRNA expression of *ATM* correlated with longer MFS in the entire cohort (*p* = 0.022, Log Rank), and in luminal-B-like tumors (*p* = 0.036). Lower mRNA expression of BLM was associated with favorable outcomes (*p* = 0.011, Log Rank), particularly in triple-negative eBC (*p* = 0.030, Log Rank). Finally, high levels of *WRN* mRNA expression correlated with significantly longer MFS compared to low mRNA expression levels (*p* = 0.009, Log Rank).

**Conclusions:**

This study underscores the prognostic significance of moderate penetrance breast cancer risk variant genes, such as *ATM*, *BLM*, and *WRN*, for survival outcomes in eBC.

## 1 Introduction

Advances in research using microarray-based analysis enhance our ability to predict better, improve patients’ outcomes, and personalize their treatment. Knowledge about gene expression profiling has led to the classification of breast cancer into four molecular subtypes with different disease outcomes Haibe-Kains et al. [1]. Further studies have investigated the prognostic impact of messenger ribonucleic acid (mRNA) expression of several genes on patients with early-stage breast cancer (eBC) [2–4]. However, research on deoxyribonucleic acid (DNA) repair gene expression, such as non*-Breast Cancer (BRCA)* genes and their impact on the prognosis of eBC patients, remains limited.

Building on our previous findings on the mRNA expression of *BRCA1, BRCA2*, and *Partner and localizer of BRCA2 (PALB2)*, we further examined the impact of 20 non*-BRCA* genes on survival in a cohort of 461 eBC patients, using Kaplan-Meier curves and Cox regression analyses [5]. In the present study, we investigated the predictive significance of *Ataxia-telangiectasia mutated (ATM), Bloom helicase gene (BLM)*, and *WRN RecQ-Like Helicase (WRN)* mRNA expression.

*ATM* is a tumor suppressor gene that plays a key role in cell cycle control, apoptosis, and oxidative stress [6]. Recently published studies have shown that certain variants of *ATM* are linked to a higher risk of developing breast cancer and poor prognosis [7,8]. Further studies have investigated the impact of mRNA expression of *ATM* on the survival of patients with eBC [9–11].

*BLM* and *WRN* belong to the RecQ DNA helicase family, which plays a critical role in protecting and stabilizing the genome [12]. Since their prognostic role in eBC remains unknown, a few studies aimed to investigate the prognostic impact of mRNA expression of the RecQ family [13]. *BLM* plays an important role in replication and proliferation, and it is not surprising that *BLM* mRNA overexpression is more commonly observed in eBC with a worse prognosis [13]. *WRN*, a known tumor suppressor, plays an important role in DNA repair, replication, recombination, and maintenance of nuclear telomeres. Reduced *WRN* expression leads to genomic instability [14,15]. Preclinical studies have also shown that expression of *WRN* in eBC cell lines inhibits tumor growth in mouse xenograft models [16].

Microarray analysis presents a modality to measure gene expression signatures, which can then be used as a component of genomic-based prognostic tests. Furthermore, expression profiling of DNA repair genes could help stratify patients for future clinical trials investigating targeted therapies. Thus, we provide crucial insights regarding the prognostic significance and the potential clinical relevance of mRNA expression of *non-BRCA* genes such as *ATM, BLM*, and *WRN* in patients with eBC. Such tests can help identify subgroups of eBC patients with worse prognosis and integrate their role in modern diagnostic and prognostic models.

## 2 Materials and Methods

### 2.1 Patient’s Characteristics

This study was conducted in a consecutive cohort of 461 patients with eBC and long-term follow-up, treated at the Department of Obstetrics and Gynecology, University Medical Center Mainz, between 1986 and 2000. eBC was defined as tumors classified as T1–T3 with N0–N1 disease, as well as high-risk eBC cases (T4 or N2). The cohort comprised all patients with eBC who showed no evidence of distant metastasis at diagnosis. Patients presenting with metastatic disease or lacking adequate clinical information and/or mRNA expression data were excluded. Written informed consent was obtained from all participants, and all procedures adhered to ethical and legal standards consistent with the Declaration of Helsinki. The study protocol was approved by the Institutional Review Board of the Ethics Committee of Rheinland-Pfalz, Germany (No. 837.139.05 [4797], 20 October 2005).

Clinicopathological information was retrieved from pathology records and the institutional breast cancer database. Collected data included age at diagnosis, histological grade, tumor size, nodal status, estrogen receptor (ER) and progesterone receptor (PR) status, human epidermal growth factor receptor 2 (HER2) status, Ki-67 index, protocols of administered treatments, and follow-up outcomes. Of the full cohort, 200 node-negative patients did not receive any adjuvant therapy following surgery. Adjuvant tamoxifen was administered to 165 patients, whereas 96 patients received adjuvant chemotherapy. As all treatments were administered postoperatively and Affymetrix microarray analyses were performed on fresh-frozen primary tumor tissue, these therapies did not influence the measured mRNA expression profiles. The clinical characteristics of this cohort have been described previously [5].

### 2.2 Gene-Expression Analyses

Fresh-frozen breast tumor samples were collected from the Department of Obstetrics and Gynecology, University Medical Center Mainz and analyzed using HG-U133A microarrays (Affymetrix, Santa Clara, CA, USA) to assessrelative transcript levels in breast cancer tissue, as described in previous results [3,5,17].

Tumor samples were snap-frozen and stored at −80°C withtumor cell content exceeding 40% in all samples. Approximately 50 mg of frozen breast tumor tissue was crushed in liquid nitrogen. RLT buffer was added to the tissue, and the homogenate was processed through a QIAshredder column (Qiagen, Hilden, Germany). Total RNA was isolated with the RNeasy Kit (Qiagen, Hilden, Germany, Qiagen Kit: Cat no./ID. 74106), following the manufacturer’s instructions.

RNA yield was quantified by UV absorbance, and RNA quality was assessed via rRNA band integrity using an Agilent 2100 Bioanalyzer with a RNA 6000 LabChip kit (Agilent Technologies, Santa Clara, CA, USA). From 5 μg of total RNA, labeled cRNA was synthesized using Roche Microarray cDNA Synthesis, Microarray RNA Target Synthesis (T7), and Microarray Target Purification kits (Roche Applied Science, Mannheim, Germany), according to the manufacturer’s instructions.

Microarray Data Processing and Normalization

Raw expression data (CEL files) were normalized using frozen robust multiarray analysis (fRMA), with global scaling applied to achieve a mean target intensity of 500. Samples exhibiting suboptimal signal intensities (scaling factors > 25) or high glyceraldehyde-3-phosphate dehydrogenase (GAPDH) 3^′^/5^′^ ratios (>5) were relabeled and rehybridized on new arrays.

For the gene expression analysis, data derived from fresh frozen tissue and measured using HG-U133A arrays are reported using TGT500 scaling. The complete dataset of 461 samples, along with updated follow-up information, has been deposited in the NCBI GEO database under accession number GSE158309 (https://www.ncbi.nlm.nih.gov/geo/query/acc.cgi?acc=GSE158309).

The following single genes were considered for this gene expression analysis (Table 1).

**Table 1:** Genes included in the study and corresponding probe sets

Genes	Probesets
*TP53*	201746_at, 211300_s_at
*PTEN*	204053_x_at, 204054_at, 211711_s_at, 217492_s_at, 222176_at
*PTEN1*	204053_x_at, 204054_at, 211711_s_at, 217492_s_at
*ATM*	212672_at, 208442_s_at, 210858_x_at
*CHEK2*	210416_s_at
*BARD1*	205345_at
*ATR*	208531_at, 209902_at, 209903_s_at, 220092_s_at, 220093_at
*BAP1*	201419_at, 205215_at, 206144_at
*BLM*	205733_at
*BRIP1*	235609_at, 221703_at
*CHEK1*	205393_s_at, 205394_at, 238075_at
*FANCA*	203805_s_at, 203806_s_at, 215530_at
*FANCC*	205189_s_at
*FANCF*	218689_at
*MRE11A*	205395_s_at, 211334_at
*NBN*	202905_x_at, 202906_s_at, 202907_s_at, 207675_x_at, 210237_at, 216052_x_at, 217299_s_at
*RAD50*	208393_s_at, 209349_at
*RAD51C*	206066_s_at, 209849_s_at
*RAD51D*	209965_s_at, 37793_r_at
*WRN*	205667_at

The metagenes of *tumor protein p53* (TP53), *phosphatase and tensin homolog* (*PTEN*), *Ataxia Telangiectasia Mutated* (*ATM*), *checkpoint kinase 2* (*CHEK2*), *BRCA1-Associated Ring Domain protein 1* (*BARD1*), *ATR serine/threonine kinase* (*ATR*), *BRCA1 associated protein 1* (*BAP1*), *Bloom helicase* (*BLM*), *BRCA1 Interacting Helicase 1* (*BRIP1*), *checkpoint kinase 1* (*CHEK1*), *Fanconi anemia complementation group A* (*FANCA*), *NIBRIN* (*NBN*), *Fanconi anemia complementation group C* (*FANCC*), *Fanconi anemia complementation group F* (*FANCF*), *Meiotic recombination 11A* (*MRE11A*), *RAD50 Double Strand Break Repair Protein* (*RAD50*), *RAD51 paralog C* (*RAD51C*), *RAD51 paralog D* (*RAD51D*), *WRN RecQ Like Helicase* (*WRN*) were calculated for each sample as the average expression of all genes in the gene cluster. As mentioned earlier, the median mRNA expression was used as the cut-off value. All patients included in this study were subdivided into two groups based on low and high mRNA expression levels.

### 2.3 Statistical Analysis

Statistical analyses were conducted using SPSS software, version 27.0 (SPSS Inc., Chicago, IL, USA). We performed Kaplan–Meier survival analysis as well as univariable and multivariable Cox proportional hazards regression models to evaluate the prognostic relevance of mRNA expression of the above-mentioned genes with respect to metastasis-free survival (MFS) in the overall cohort. MFS was defined as the interval between initial diagnosis and the occurrence of distant metastasis.

Subgroup analyses were performed to assess differences in mRNA expression levels across molecular subtypes. The multivariable Cox models were adjusted for tumor stage, histological grade, lymph node status, and Ki-67 index. Survival distributions were compared using the log-rank test, and statistical significance was defined as a two-sided *p*-value < 0.05. Post hoc Dunn’s tests with Bonferroni correction were applied to identify specific pairwise differences in gene expression. In addition, survival analyses were repeated in the subgroup of patients who did not receive any systemic therapy (N = 200).

The primary endpoint of the study was the association between mRNA expression levels and MFS. Secondary endpoints included the relationships between median mRNA expression levels of *ATM*, *BLM*, and *WRN* and both patient-related and tumor-related characteristics.

### 2.4 Validation of mRNA Expression of ATM, BLM, and WRN in Independent Cohorts

**Figure A1:**
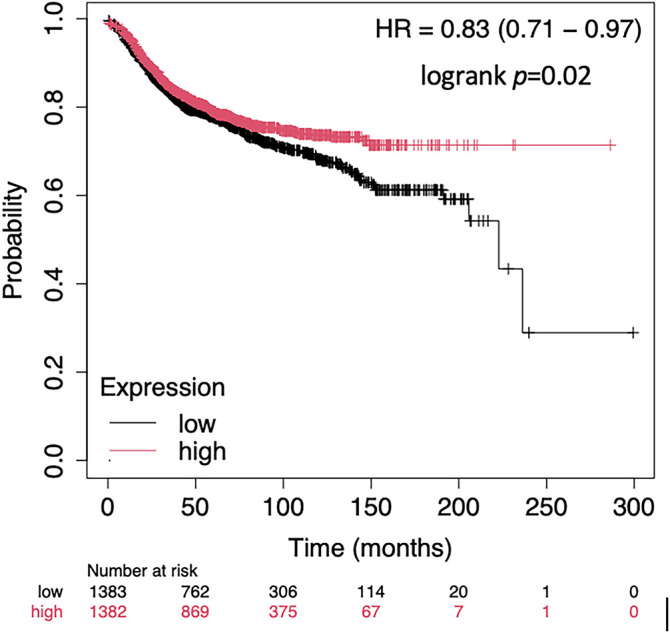
Kaplan Meier Plot of the *ATM* mRNA expression in terms of MFS of an unselected breast cancer cohort using publicly available gene expression data [18]

**Figure A2:**
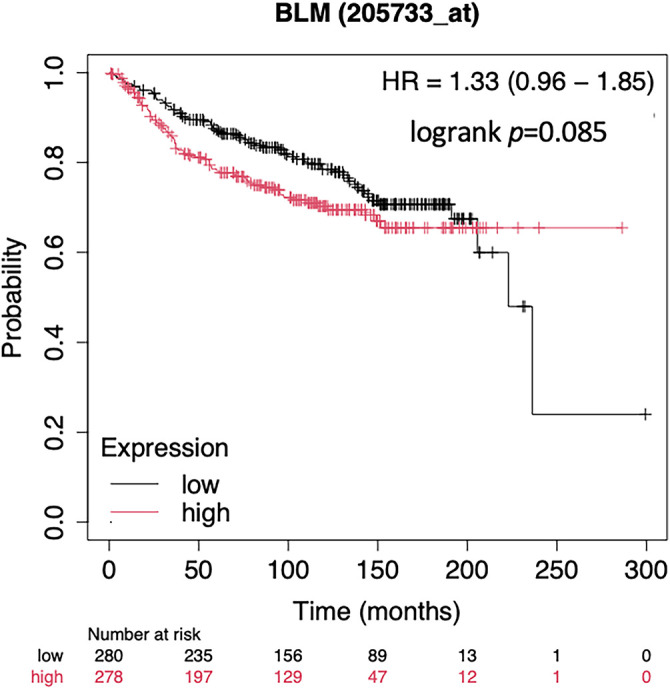
Kaplan Meier Plot of the *BLM* mRNA expression in terms of MFS of an unselected breast cancer cohort using publicly available gene expression data [18]

**Figure A3:**
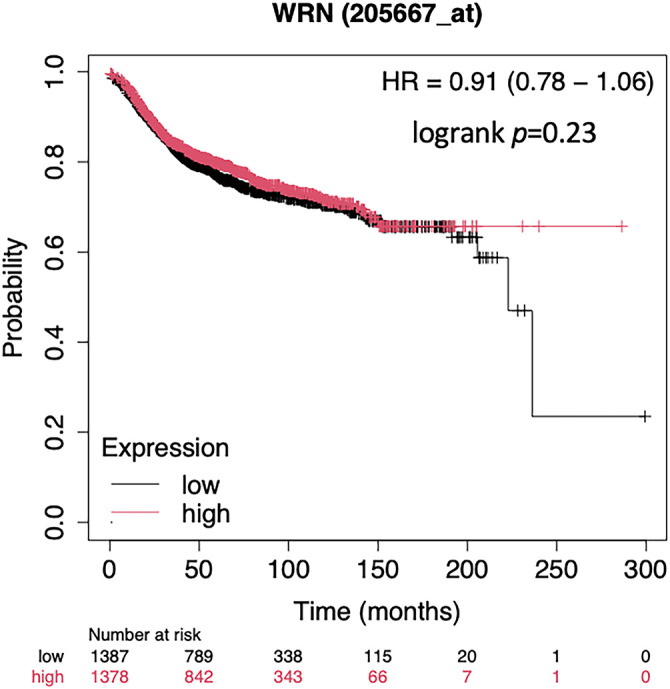
Kaplan Meier Plot of the *WRN* mRNA expression in terms of MFS of an unselected breast cancer cohort using publicly available gene expression data [18]

We utilized the Kaplan-Meier Plotter database (http://kmplot.com/analysis) to validate our results on a larger, independent cohort among all samples of eBC and within various intrinsic subtypes and clinicopathological characteristics. The prognostic significance of *ATM* (*p* = 0.022, Log Rank), was successfully validated in previously published datasets. In contrast, the prognostic impact of *BLM* and *WRN* could not be validated in the same datasets (Appendix A, Figs. A1–A3) [18].

### 2.5 Subgroup Analyses

Intrinsic subtypes were determined according to the classification proposed by Haibe-Kains et al., which is based on the expression of ESR1, HER2, and AURKA [1].

Survival analyses were subsequently conducted within the major molecular breast cancer subgroups. Tumors classified as Luminal A–like were characterized by ESR1 positivity, absence of HER2 overexpression, and low proliferative activity (low AURKA expression). Luminal B–like tumors were also ESR1-positive and HER2-negative but showed high proliferation (high AURKA expression). The HER2-positive group consisted of tumors demonstrating HER2 amplification or overexpression. The Basal-like subtype was defined by the lack of both ESR1 and HER2 expression.

## 3 Results

The effect of mRNA expression levels of 20 non*-BRCA* susceptibility genes on patient survival was evaluated in a cohort of eBC patients with long-term follow-up (median 147 months, ranging from 1 to 306 months). Only three genes (*ATM*, *BLM*, *WRN*) were identified in our analyses for association with MFS. The identified associations remained significant after Bonferroni correction.

### 3.1 The Prognostic Effect of mRNA Expression of 20 Genes in Patients with eBC

As shown in Table 1, the prognostic significance of non-BRCA susceptibility genes’ mRNA expression was assessed using the available data. *ATM* mRNA expression levels were significantly associated with MFS (Hazard Ratio, HR 0.672; 95% Confidence Interval, CI 0.477–0.948; *p* = 0.023). Interestingly, even mRNA expression of *BLM* and *WRN*, known as *RecQ deoxyribonucleic acid (DNA) helicase* family members, demonstrated a significant impact on MFS in the whole cohort (*p* < 0.05). In contrast, mRNA expression of the remaining genes showed no significant association with MFS (all *p* > 0.05; Table 2).

**Table 2:** Association between mRNA-expression of 20 genes and metastasis-free survival using Cox regression analysis

Gene-level expression	HR	95% CI	*p*-value
*TP53* Metagene	1.038	0.771–1.396	0.806
*PTEN* Metagene	0.721	0.511–1.016	0.062
*PTEN_1* Metagene	0.736	0.522–1.038	0.081
*ATM* Metagene	0.672	0.477–0.948	0.023
*CHEK2*	0.859	0.611–1.207	0.380
*BARD1*	1.362	0.967–1.917	0.077
*ATR* Metagene	1.242	0.882–1.748	0.215
*BAP1* Metagene	0.966	0.688–1.357	0.842
*BLM*	1.560	1.105–2.203	0.012
*BRIP 1*	1.136	0.809–1.597	0.462
*CHEK1* Metagene	1.106	0.787–1.554	0.563
*FANCA* Metagene	1.405	0.997–1.981	0.052
*NBN* Metagene	0.923	0.657–1.297	0.644
*FANCC*	1.173	0.834–1.649	0.359
*FANCF*	0.891	0.634–1.252	0.505
*MRE11A* Metagene	0.983	0.700–1.382	0.923
*RAD 50* Metagene	0.800	0.568–1.125	0.199
*RAD 51C* Metagene	1.130	0.804–1.588	0.482
*RAD 51D* Metagene	1.125	0.801–1.581	0.496
*WRN*	0.635	0.450–0.897	0.010

Note: *TP53, tumor protein p53; PTEN, phosphatase and tensin homolog; ATM, Ataxia telangiectasia mutated; CHEK2, checkpoint kinase 2; BARD1, BRCA1-associated ring domain protein 1; ATR, ATR serine/threonine kinase; BAP1, BRCA1 associated protein 1; BLM, Bloom helicase; BRIP1, BRCA1 interacting helicase 1; CHEK1, checkpoint kinase 1; FANCA, Fanconi anemia complementation group A; NBN, NIBRIN; FANCC, Fanconi anemia complementation group C; FANCF, Fanconi anemia complementation group F; MRE11A, meiotic recombination 11A; RAD50, RAD50 double strand break repair protein; RAD51C, RAD51 paralog C; RAD51D, RAD51 paralog D; WRN, WRN RecQ Like Helicase*; mRNA, messenger ribonucleic acid; CI, confidence interval; HR, hazard ratio; *p*-value < 0.05 is considered significant.

### 3.2 Patients and Tumor Characteristics

The median age at initial diagnosis of the patients included in our study was 62 years (range: 30–93). Patients and tumor characteristics, as well as their association with mRNA expression of *ATM*, *BLM*, and *WRN*, are outlined in Table 3.

**Table 3:** Associations between mRNA expression of *ATM, BLM, WRN* and patients and tumor characteristics

Character istic	Patients N. (%)		*ATM* lower expression N. (%)	*ATM* higher expression N. (%)	*p*-value	*BLM* lower expression N. (%)	*BLM* higher expression N. (%)	*p*-value	*WRN* lower expression N. (%)	*WRN* higher expression N. (%)	*p*-value
**Tumor size**											
pTis	5 (1.1)	3 (1.3)	2 (0.9)			3 (1.3)	2 (0.9)		3 (1.3)	2 (0.9)	
pT1	183 (39.8)	76 (33.0)	107 (46.5)			110 (47.8)	73 (31.7)		79 (34.3)	104 (45.2)	
pT2	214 (46.5)	111 (48.3)	103 (44.8)			98 (42.6)	116 (50.4)		104 (45.2)	110 (47.8)	
pT3	19 (4.1)	12 (5.2)	7 (3.0)			6 (2.6)	13 (5.7)		18 (7.8)	1 (0.4)	
pT4	39 (8.5)	28 (12.2)	11 (4.8)		0.006	13 (5.7)	26 (11.3)	0.003	26 (11.3)	13 (5.7)	<0.001
Missing data	1 (0.2)										
**Axillary nodal status**											
pN0	253 (57.5)	98 (45.2)	155 (69.5)			143 (64.7)	110 (50.2)		111 (50.2)	142 (64.8)	
pN1	138 (31.4)	85 (39.2)	53 (23.8)			58 (26.2)	80 (36.5)		78 (35.3)	60 (27.4)	
pN2	49 (11.1)	24 (15.7)	15 (6.7)		<0.001	20 (9.0)	29 (13.2)	0.009	32 (14.5)	17 (7.8)	0.005
Missing data	21 (4.6)										
**Histological grade**											
I	62 (14.5)	21 (9.1)	42 (18.2)			53 (23.0)	10 (4.3)		22 (9.6)	41 (17.7)	
II	261 (60.8)	150 (65.2)	137 (59.3)			151 (65.7)	136 (58.9)		145 (63.0)	142 (61.5)	
III	106 (24.7)	59 (25.7)	52 (22.5)		0.018	26 (11.3)	85 (36.8)	<0.001	63 (27.4)	48 (20.8)	0.020
Missing data	32 (6.9)										
**Age at study entry**											
<50 years	95 (20.6)	49 (21.3)	55 (23.8)			46 (20.0)	58 (25.1)		50 (21.7)	54 (23.4)	
≥50 years	366 (79.4)	181 (78.7)	176 (76.2)		0.520	184 (80.0)	173 (74.9)	0.190	180 (78.3)	177 (76.6)	0.674
**Estrogen receptor status**											
Positive	252 (73.5)	148 (80.9)	104 (65.0)			129 (78.2)	123 (69.1)		122 (70.9)	130 (76.0)	
Negative	91 (26.5)	35 (19.1)	56 (35.0)		<0.001	36 (21.8)	55 (30.9)	0.057	50 (29.1)	41 (24.0)	0.285
**Progesterone receptor status**											
Positive	204 (59.5)	121 (66.1)	83 (51.9)			102 (61.8)	102 (57.3)		99 (57.6)	105 (61.4)	
Negative	139 (40.5)	62 (33.9)	77 (48.1)		0.007	63 (38.2)	76 (42.7)	0.395	73 (42.4)	66 (38.6)	0.468
**Ki-67 (%) median**											
≤20	199 (59.9)	80 (55.6)	119 (63.3)			135 (78.5)	64 (40.0)		84 (55.3)	115 (63.9)	
>20	133 (40.1)	64 (44.4)	69 (36.7)		0.154	37 (21.5)	96 (60.0)	<0.001	68 (44.7)	65 (36.1)	0.110
Missing data	129 (28.0)										
**Molecular subtype**											
Luminal A-like	189 (41.0)	71 (30.9)	118 (51.1)			150 (65.2)	39 (16.9)		85 (37.0)	104 (45.0)	
Luminal B-like	182 (39.5)	117 (50.9)	65 (28.1)			65 (28.3)	117 (50.6)		95 (41.3)	87 (37.7)	
Her2-positive	39 (8.5)	18 (7.8)	21 (9.1)			11 (4.8)	28 (12.1)		25 (10.9)	14 (6.1)	
Basal-like	51 (11.1)	24 (10.4)	27 (11.7)		<0.001	13 (25.5)	47 (20.3)	<0.001	25 (10.9)	26 (11.3)	0.146

Note: *ATM*, *Ataxia telangiectasia mutated*; *BLM*, *Bloom helicase gene*; *WRN*, *WRN RecQ Like Helicase*; *p*-value < 0.05 is considered significant, N (%): indicates the proportion of patients within the group shown in the column.

### 3.3 Impact of ATM mRNA Expression on the Survival of eBC Patients

**Figure 1:**
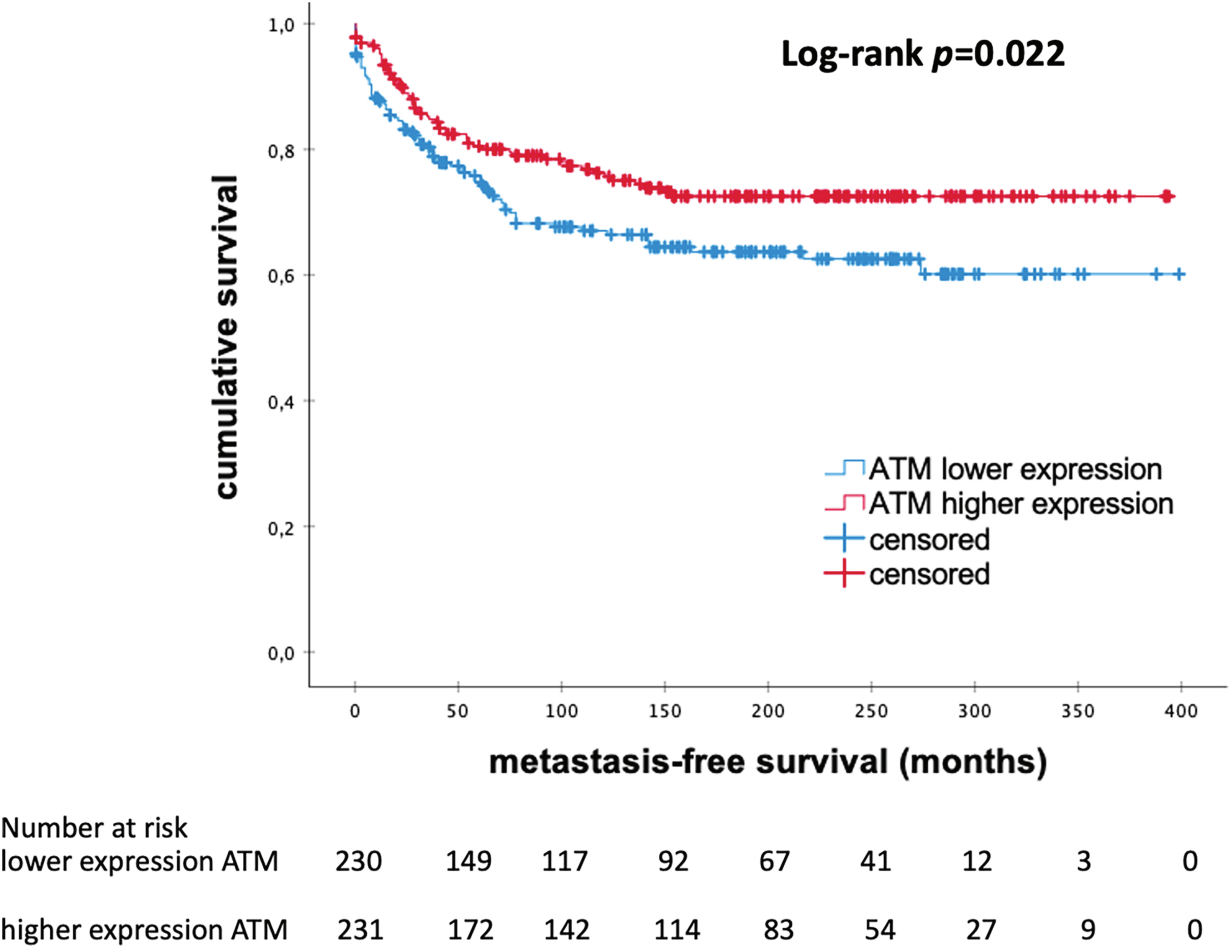
Kaplan–Meier analysis of MFS in patients with eBC according to levels of ATM mRNA expression. *ATM*, *Ataxia Telangiectasia Mutated*; *p*-value < 0.05 is considered significant

Kaplan-Meier survival analysis indicated that high mRNA expression of ATM correlated with longer MFS in the whole cohort (*p* = 0.022; Fig. 1). The median MFS was 148.0 and 103.0 months in each subgroup, respectively. In a multivariate Cox regression analysis, tumor size and histological grading were identified as independent prognostic factors. The multivariate Cox regression analysis, presented in Table 4 shows that ATM mRNA expression levels did not maintain independent significance after adjusting for other clinical variables (HR 0.913; 95% CI 0.579–1.441; *p* = 0.697).

**Table 4:** Univariable and multivariable Cox regression analysis of ATM mRNA expression for MFS adjusted for age, tumor size, grade of differentiation, lymph node status and the proliferation marker Ki-67

Variables		Univariable model HR (95% CI)	*p*-value	Multivariable model HR (95% CI)	*p*-value
*ATM*	High vs. low	0.672 (0.477–0.948)	0.023	0.913 (0.579–1.441)	0.697
Age	<50 vs. ≥50	0.838 (0.569–1.234)	0.371		
Tumor size	T2–4 vs. T1	1.839 (1.543–2.193)	<0.001	1.532 (1.164–2.016)	0.002
Histological grade of differentiation	GIII vs. GI/II	2.386 (1.221–3.305)	<0.001	2.014 (1.369–2.963)	<0.001
Axillary nodal status	N1, 2, 3 vs. N0	1.939 (1.520–2.473)	<0.001	1.191 (0.846–1.678)	0.316
Ki-67	>20% vs. <20%	1.878 (1.220–2.890)	0.004	1.279 (0.820–1.997)	0.278

Note: *ATM*, *Ataxia telangiectasia mutated*; *p*-value < 0.05 is considered significant, CI, confidence interval; HR, hazard ratio.

### 3.4 Impact of ATM mRNA Expression on Survival among Different Molecular Subtypes of eBC

**Figure 2:**
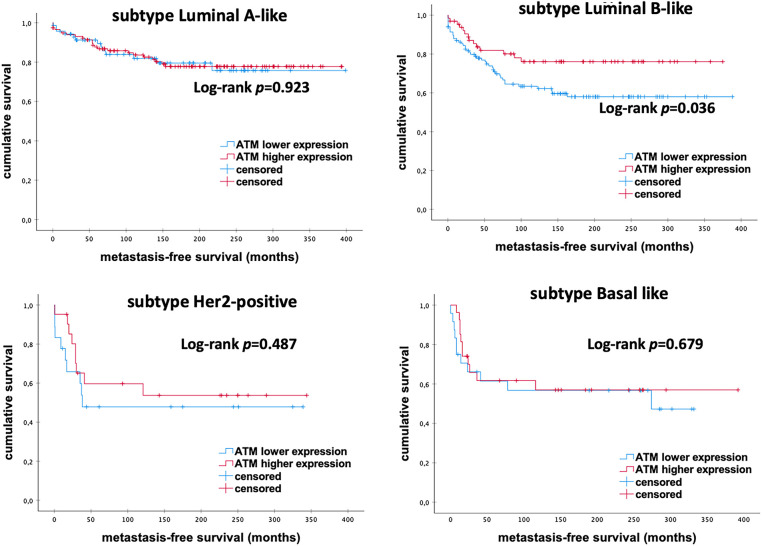
Kaplan–Meier analysis of MFS in eBC patients among different intrinsic molecular subtypes according to *ATM* mRNA expression levels. *ATM*, *Ataxia Telangiectasia Mutated*; *p*-value < 0.05 is considered significant

Further, subgroup analyses demonstrated a significant impact on MFS in patients with Luminal-B-like eBC. Kaplan Meier analysis indicated that elevated mRNA expression levels of *ATM* correlate with longer MFS in Luminal-B-like subtype (*p* = 0.036; Fig. 2). No significant difference was noticed in other molecular subtypes (all *p* > 0.050). These findings suggest that high mRNA expression levels of *ATM* are associated with better outcomes, especially in patients with Luminal-B-like eBC.

### 3.5 Impact of BLM mRNA Expression on the Survival of eBC Patients

**Figure 3:**
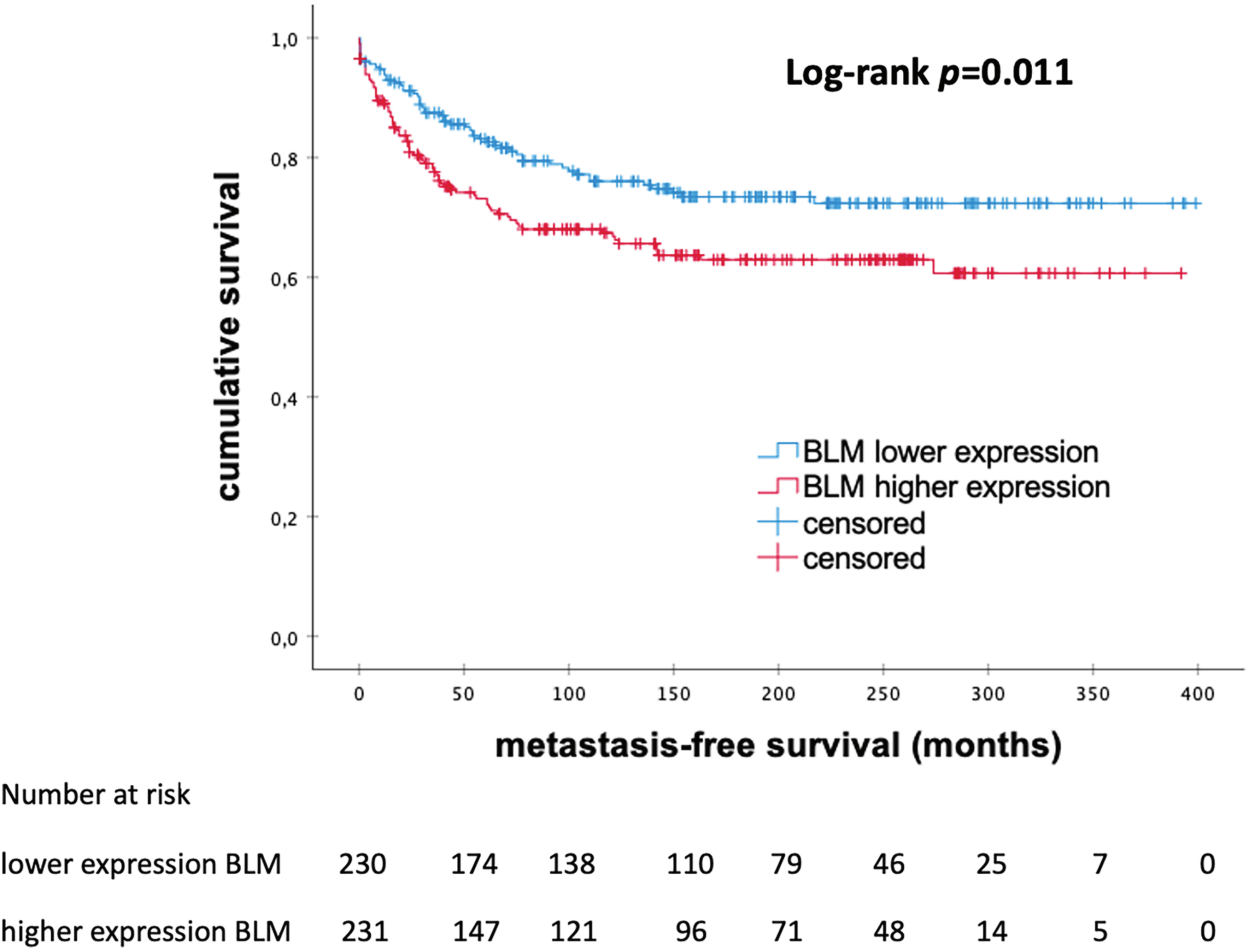
Kaplan–Meier analysis of metastasis-free survival in patients with early breast cancer according to the levels of *BLM* mRNA expression, *BLM*, *Bloom helicase*; *p*-value < 0.05 is considered significant

The Kaplan–Meier analysis showed a significant prognostic effect on the levels of BLM mRNA expression among eBC patients. There was a trend in the entire cohort towards a favorable outcome associated with lower mRNA expression of BLM (*p* = 0.011; Fig. 3). The median MFS was 146.0 months in the subgroup with low levels of BLM expression and 111 months in the high BLM expression group.

The multivariate Cox regression analysis for the BLM mRNA expression (Table 5) indicates that the levels of BLM mRNA expression did not retain independent significance when adjusted for other clinical variables.

**Table 5:** Univariable and multivariable Cox regression analysis of BLM mRNA expression for MFS adjusted for age, tumor size, grade of differentiation, lymph node status and the proliferation marker Ki-67

Variables		Univariable model HR (95% CI)	*p*-value	Multivariable model HR (95% CI)	*p*-value
*BLM*	High vs. low	1.560 (1.105–2.203)	0.012	0.817 (0.495–1.348)	0.428
Age	<50 vs. ≥50	0.861 (0.585–1.268)	0.448		
Tumor size	T2–4 vs. T1	1.817 (1.529–2.161)	<0.001	1.517 (1.156–1.991)	0.003
Histological grade of differentiation	GIII vs. GI/II	2.321 (1.700–3.169)	<0.001	2.151 (1.437–3.220)	<0.001
Axillary nodal status	N1, 2, 3 vs. N0	1.940 (1.527–2.465)	<0.001	1.249 (0.892–1.747)	0.195
Ki-67	>20% vs. <20%	1.814 (1.136–2.898)	0.013	1.290 (0.817–2.039)	0.275

Note: *BLM*, *Bloom helicase*; *p*-value < 0.05 is considered significant, CI, confidence interval; HR, hazard ratio.

### 3.6 Impact of BLM mRNA Expression on Survival among Different Molecular Subtypes of eBC

**Figure 4:**
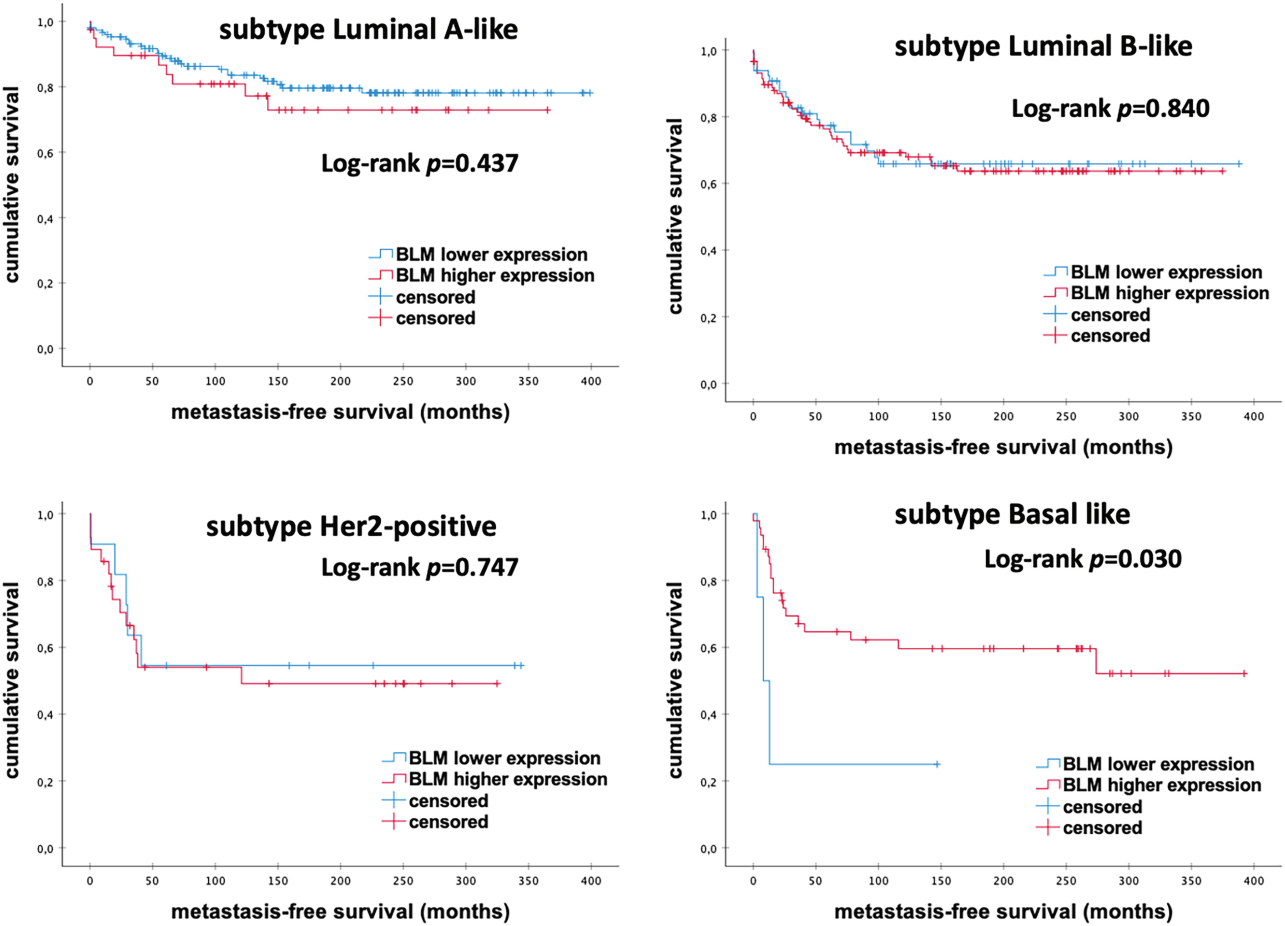
Kaplan–Meier analysis of MFS in eBC patients among different intrinsic molecular subtypes according to the levels of *BLM* mRNA expression. *BLM*, *Bloom helicase*; *p*-value < 0.05 is considered significant

Overall, we detected an association of low *BLM* mRNA expression with better prognosis in eBC patients (Fig. 3). The level of *BLM* mRNA expression differed between different intrinsic subtypes of eBC. Stratified subgroup analyses revealed significant survival differences, surprisingly in favor of high mRNA expression among Basal-like tumors. However, the power of the analysis is limited by the very small sample size (*p* = 0.030; Fig. 4).

### 3.7 Impact of WRN mRNA Expression on the Survival of eBC Patients

**Figure 5:**
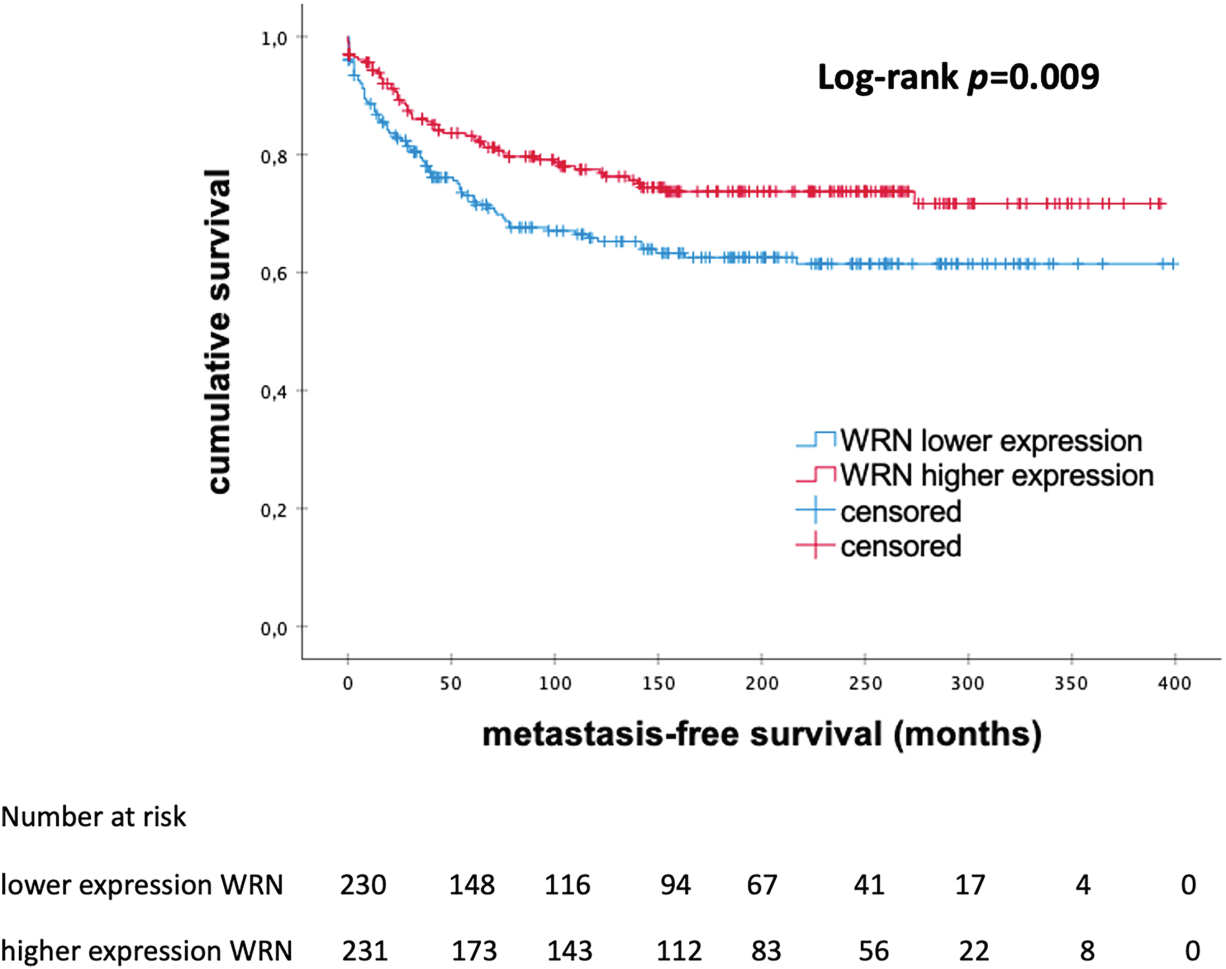
Kaplan–Meier analysis of MFS in patients with eBC according to the levels of *WRN* mRNA expression. *WRN*, *WRN RecQ Like Helicase*; *p*-value < 0.05 is considered significant

The Kaplan–Meier analysis showed that patients with high levels of *WRN* mRNA expression had significantly longer MFS than those with low mRNA expression (145.0 vs. 102.5 months; *p* = 0.009) (Fig. 5).

In the multivariable analysis, mRNA expression of *WRN* could not be identified as an independent prognostic factor (HR 0.962; 95% CI 0.606–1.527; *p* = 0.871). As shown in Table 6, tumor size and grading were identified as independent prognostic factors.

**Table 6:** Univariable and multivariable Cox regression analysis of *WRN* mRNA expression for MFS adjusted for age, tumor size, grade of differentiation, lymph node status and the proliferation marker Ki-67

Variables		Univariable model HR (95% CI)	*p*-value	Multivariable model HR (95% CI)	*p*-value
*WRN*	High vs. low	0.635 (0.450–0.897)	0.010	0.962 (0.606–1.527)	0.871
Age	<50 vs. ≥50	0.840 (0.571–1.238)	0.379		
Tumor size	T2–4 vs. T1	1.807 (1.517–2.154)	<0.001	1.549 (1.176–2.042)	0.002
Histological grade of differentiation	GIII vs. GI/II	2.286 (1.708–3.059)	<0.001	2.205 (1.377–2.979)	<0.001
Axillary nodal status	N1, 2, 3 vs. N0	1.934 (1.523–2.457)	<0.001	1.233 (0.883–1.722)	0.219
Ki-67	>20% vs. <20%	1.707 (1.152–2.528)	0.008	1.254 (0.803–1.958)	0.320

Note: *WRN*, *WRN RecQ Like Helicase*; CI, confidence interval; HR, hazard ratio; *p*-value < 0.05 is considered significant.

### 3.8 Impact of WRN mRNA Expression on Survival among Different Molecular Subtypes of eBC

**Figure 6:**
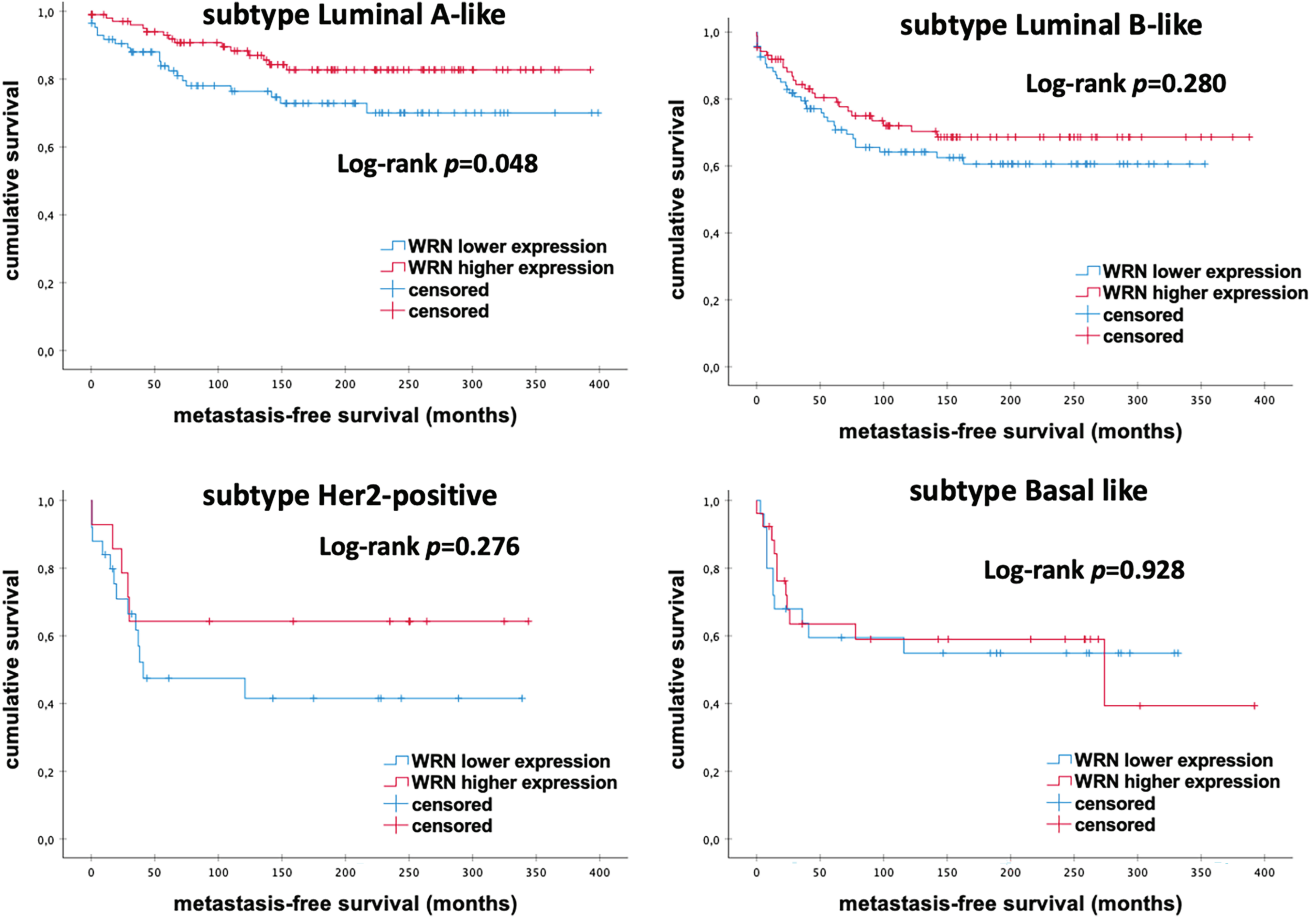
Kaplan–Meier analysis of MFS in eBC patients among different intrinsic molecular subtypes according to the levels of *WRN* mRNA expression. *WRN*, *WRN RecQ Like Helicase*; *p*-value < 0.05 is considered significant

As shown in Fig. 6, further subgroup analyses revealed a significant increase in MFS rates in patients with Luminal-A-like eBC and high levels of *WRN* mRNA expression (*p* = 0.048). However, there were no significant differences in MFS among other molecular subtypes (all *p* > 0.050).

### 3.9 The Prognostic Effect of mRNA Expression of ATM, BLM and WRN Genes in eBC Patients without Adjuvant Therapy

We performed further survival analysis in order to investigate the prognostic impact of the mRNA Expression of *ATM, BLM* and *WRN* in eBC patients (N = 200), who did not receive any adjuvant therapy. Similar to the entire cohort of patients, even within the untreated cohort, we demonstrated that the mRNA expression level was significantly associated with MFS in Kaplan-Meier and univariable Cox regression analyses (Table 7).

**Table 7:** Association between mRNA-expression of *ATM*, *BLM* and *WRN* and MFS using Cox regression univariable analysis in the adjuvant non-treated group of patients

Gene-Level Expression	HR	95% CI	*p*-value
*ATM*	0.497	0.282–0.878	0.015
*BLM*	1.560	1.105–2.203	0.011
*WRN*	0.635	0.450–0.897	0.009

Note: *ATM*, *Ataxia Telangiectasia Mutated*; *BLM*, *Bloom helicase*; *WRN*, *WRN RecQ Like Helicase*; mRNA, messenger ribonucleic acid; CI, confidence interval; HR, hazard ratio; *p*-value < 0.05 is considered significant.

In the multivariable analysis, mRNA expression of *ATM, BLM* and *WRN* could not be identified as an independent prognostic factor. The Kaplan-Meier curves can be found as Supplementary Material Figs. S1–S3.

## 4 Discussion

Gene expression analyses in eBC have shown that mRNA expression levels of particular genes are strong independent prognostic biomarkers. In this study, we examined the expression of mRNA levels of 20 *non-BRCA* genes in a cohort of 461 patients with eBC to assess their prognostic impact. Among the 20 analyzed genes, only the mRNA expression of *ATM, BLM* and *WRN* significantly correlated with prognosis in eBC patients. The other non-*BRCA* genes exhibited no predictive value for MFS.

### 4.1 The Prognostic Role of ATM mRNA Expression and Its Clinical Impact

Previous studies have established that *ATM* plays a crucial role in the development and progression of early breast cancer (eBC). Notably, down-regulated *ATM* mRNA expression has been observed in breast cancer tissues [9,19,20]. However, limited clinical studies have investigated the prognostic significance of *ATM* expression levels in eBC. Our study indicates that patients with higher *ATM* expression levels tend to have better survival rates compared to those with lower levels, particularly within the Luminal-B-like eBC subgroup. These findings align with the results of a study. By Ye et al., which involved 471 eBC patients and demonstrated that high *ATM* mRNA expression correlates with improved disease-free survival (DFS) (HR 0.66; 95% CI 0.36–1.24) and overall survival (OS) (HR 0.80; 95% CI 0.42–1.51) compared to low *ATM* mRNA expression [9]. Similarly, Bueno et al. found that high *ATM* expression is associated with better prognosis in eBC (HR 0.554; 95% CI 0.3991–0.784) [10]. Furthermore, Rondeau et al. confirmed *ATM* expression serves as an independent prognostic marker in breast cancer (N = 454 patients). Their study showed that low levels of *ATM* protein were linked to poorer MFS (*p* < 0.001). Patients with lower *ATM* expression exhibited a 5-year MFS of 70.4 ± 2.5% and a 10-year MFS of 59.0 ± 2.8%. In contrast, those with higher *ATM* expression had significantly better 5-year (85.7 ± 3.2%) and 10-year (78.7 ± 3.9%) [19].

### 4.2 The Prognostic Role of BLM mRNA Expression and Its Clinical Impact

Alterations in the mRNA expression of *BLM* and *WRN*, both members of the RecQ family, have also been linked to the prognosis of eBC [21,22]. Multiple studies have shown a correlation between expression of the five RECQL genes and breast cancer tumorigenesis [23–25].

Notably, there is increasing evidence regarding the impact of *BLM* mutations on eBC prognosis [13,25]. Arora et al. investigated *BLM* mRNA expression in the Molecular Taxonomy of Breast Cancer International Consortium cohort, which included 1650 breast tumors [25]. This study was the first to demonstrate, within a large cohort, that *BLM* mRNA overexpression is associated with poor breast cancer-specific survival (*p* < 0.001), supporting the role of *BLM* as a potential biomarker for eBC. At the protein level, *BLM* also independently influenced eBC survival; altered subcellular localization and high cytoplasmic *BLM* were linked to aggressive phenotypes [25].

Similarly, Zhu et al. reported that increased *BLM* expression is associated with reduced distant MFS across all breast cancer patients [13]. Their conclusions were based on the analysis of prognostic values of *RecQ*-family mRNA expression in various intrinsic subtypes of breast cancer, utilizing the Kaplan–Meier Plotter database (http://kmplot.com/analysis).

Supporting these findings, our study demonstrates a significant correlation between increased mRNA expression of *BLM* and poor MFS through both Kaplan-Meier and Cox-regression univariable analyses. Additionally, high *BLM* mRNA levels were associated with aggressive clinicopathologic characteristics such as larger tumor size, positive axillary node status, and higher histological grade of differentiation. Given *BLM*’s proposed role in replication and proliferation, it is not surprising that high *BLM* mRNA levels were more frequently observed in eBC cases with a worse prognosis [26]. Subgroup analysis among different molecular subtypes revealed that low *BLM* mRNA expression was linked to poor prognosis, specifically in the triple-negative subgroup. These results are consistent with Arora et al.’s findings, which indicated a significant survival advantage associated with high mRNA expression in the ER-negative subgroup (*p* = 0.049) [25].

Moreover, overexpression of *BLM* and *WRN* may contribute to increased resistance of cancer cells to conventional chemotherapy [23]. Another study confirmed the relationship between *BLM* expression levels and platinum sensitivity; specifically, it examined platinum-sensitive compared to platinum-resistant triple-negative (Basal-like) eBC in two cohorts of patients receiving neoadjuvant cisplatin treatment [27]. Using an integrated genomic approach that combined differential analysis of gene expression and DNA copy number, Birkbak et al. demonstrated that overexpression of *BLM* enhances sensitivity to cisplatin. This indicates that *BLM* expression levels may serve as a valuable biomarker for predicting platinum sensitivity in Basal-like breast cancer [27].

### 4.3 The Prognostic Role of WRN mRNA Expression and Its Clinical Impact

Given the essential role of *WRN* in DNA repair and replication, several studies have investigated the prognostic potential of *WRN* expression levels in patients with early-stage breast cancer (eBC) [24]. Shamanna et al. investigated mRNA levels of *WRN* and *Topoisomerase 1 (TOP1)* in the Molecular Taxonomy of Breast Cancer International Consortium (METABRIC) cohort and found that low *WRN* mRNA expression (16.5% of tumors) was significantly associated with aggressive clinicopathological characteristics such as: high histological grade, larger tumor size, high risk Nottingham prognostic index (NPI) > 3.4, Her2 over expression, ER- and PR-tumors as well as poor DFS (*p* < 0.05) [28].

Similarly, Zhu et al. demonstrated in their study using the Kaplan–Meier plotter database to assess the prognostic value of mRNA expression of the five RecQ DNA helicase genes in connection with clinical outcomes in women with eBC that increased *WRN* mRNA expression correlates with improved overall survival (OS) (HR 0.76; 95% CI 0.61–0.94; *p* = 0.011) and enhanced relapse-free survival (RFS) (HR 0.81; 95% CI 0.73–0.91; *p* < 0.001) [13]. In ER-negative eBC patients, higher *WRN* mRNA expression was associated with better OS (HR 0.64; 95% CI 0.4–1.02; *p* = 0.056) [13].

These findings are consistent with our own findings, which show that lower mRNA expression of *WRN* correlates with worse MFS. Further subgroup analyses revealed significantly longer MFS in patients with Luminal-A-like eBC who exhibited high levels of WRN mRNA expression. However, contrary to the findings of Zhu et al., we did not observe significant differences among other molecular subtypes.

Our study highlights the contrasting prognostic implications of BLM and WRN, despite both being members of the RecQ helicase family. This difference likely reflects their distinct roles in genome maintenance and context-dependent cellular functions.

### 4.4 Controversy in the Prognostic Impact of BLM and WRN

*BLM* overexpression has been linked to increased genomic instability, which may arise from inappropriate or excessive helicase activity that leads to replication stress. This overactivity of *BLM* could support tumor cell proliferation and survival in the face of genotoxic stress, potentially explaining its association with poor prognosis [27,29]. In contrast, *WRN* possesses both helicase and exonuclease activity, playing a crucial role in maintaining telomere integrity. The loss or reduced expression of WRN can impair DNA repair mechanisms, promote chromosomal abnormalities, and accelerate tumor progression [30,31]. Additionally, WRN deficiency has been associated with microsatellite instability and could impact immune surveillance, potentially resulting in more aggressive tumors [32,33]. Thus, the contrasting prognostic roles of *BLM* and *WRN* may reflect a complex balance: overactive repair mechanisms (as seen with *BLM*) may promote the survival of genetically unstable tumor cells, while the loss of essential repair pathways (as with *WRN*) can increase genomic damage and drive tumor evolution.

Tumors with high *BLM* expression may demonstrate resistance to DNA-damaging agents due to their enhanced repair capabilities, suggesting that patients might benefit from alternative treatment regimens or combination therapies [27]. Conversely, the loss of *WRN* expression may make cells more sensitive to genotoxic chemotherapy, indicating that *WRN* levels could serve as a predictive marker for treatment response [16,34].

### 4.5 Strengths, Limitations and the Association with New Biomarkers

Our study has several strengths, including a long follow-up period and the availability of mRNA microarray data for 20 non*-BRCA* genes. This adds further weight to the observed impact of the expression levels of *ATM, BLM* and *WRN* on the survival of eBC patients. Importantly, the patients included in the study did not receive any therapy prior to surgery, which eliminates potential treatment-related effects on gene expression levels. Notably, when excluding patients who received adjuvant therapy, the results remained consistent. This suggests that the prognostic value of mRNA expression of *ATM, BLM* and *WRN* was not influenced by the adjuvant therapy. However, the study has some limitations. These include its retrospective unicentric design, a relatively small sample size, and the heterogeneity of the cohort. Additionally, there was a lack of information on *non-BRCA* gene germline mutations among the patients due to the historical nature of the study. As it was an observational study, some selection bias, including unmeasured confounders, could not be entirely avoided. In our study, the mRNA expression of *ATM, BLM* and *WRN* did not maintain its independent prognostic significance when adjusted for other clinical variables. This suggests that their impact may be context-dependent and influenced by additional factors.

One of the limitations of our study is the lack of information regarding any potential correlation between breast cancer stem cells and important biomarkers such as Cluster of Differentiation 44 (CD44), b-series ganglioside (GD2^+^) and the mRNA expression levels of *ATM, BLM* and *WRN*. Many patients with breast cancer develop resistance to chemotherapy and experience tumor recurrence, which is primarily driven by breast cancer stem cells (BCSCs) [35]. It is now well established that BCSCs, characterized by markers such as CD44^+^ and Cluster of Differentiation 24 negative/low (CD24^−^/low), as well as GD2^+^, exhibit behavior similar to that of stem cells. These cells have the ability to self-renew and differentiate into mature tumor cells, which allows the cancer to regrow and metastasize [36,37]. Currently, there is no published data directly investigating the correlation between mRNA expression levels of ATM, *BLM* and *WRN* and specific markers. However, indirect associations can be observed. For instance, high *BLM* expression is linked to poorer survival outcomes and correlates with aggressive subtypes of breast cancer, such as triple-negative breast cancer, which are known to be enriched for BCSCs characterized by the CD44^+^CD24^−^/low phenotype [38]. The diversity of BCSC populations complicates their eradication and further analysis [35].

### 4.6 Genetic Background and Clinical Impact

Guidelines for genetic testing have been established to help identify which women should undergo multigene panel testing. Both germline and somatic alterations in the *ATM, BLM* and *WRN* genes have been associated with both hereditary and sporadic cases of breast cancer [39–41]. These alterations may interact with tumor subtypes, hormone receptor statuses, and the immune landscape, further influencing prognosis and treatment responses. *ATM* plays a central role in the DNA damage response following double-strand breaks and in cell cycle checkpoint control [42,43]. Germline heterozygous mutations in the *ATM* gene, which are found in 0.7% of the population, are associated with an increased risk of breast cancer. Among these mutations, the rare missense variant c.7271T>G (p.V2424G) is linked to a particularly high breast cancer risk [44]. Female carriers of the *ATM* gene have about a twofold increased lifetime risk of developing ER-positive breast cancer, with a penetrance of 20% to 30% [41,45,46]. The *BLM* gene is currently being studied as a potential breast cancer susceptibility gene and has shown a connection to survival rates following immunotherapy across various cancers. However, clear evidence indicates that mutations in the *BLM* gene, when present in a homozygous state, increase the risk of eBC, with an average age of diagnosis around 33 years [47]. In contrast, Kluzniak et al. and other recent studies have indicated that heterozygous *BLM* mutations do not significantly increase the risk of eBC and likely do not elevate the risk of other cancers either [48]. Research indicates that WRN may be a breast cancer susceptibility gene; however, most studies are based on small cohorts, highlighting the need for validation of these findings [49,50].

We only investigated the prognostic role of mRNA expression of these genes. This study did not examine the association between their mutations and prognosis or mRNA expression.

### 4.7 Future Perspectives on the Prognostic Impact of ATM, BLM and WRN mRNA Expression

The identification of altered mRNA expression levels in DNA repair genes may not only serve as a prognostic marker but could also guide the selection of patients for targeted therapeutic interventions in eBC. However, the potential therapeutic implications of mRNA Expression of non-BRCA DNA repair genes have been investigated in only a few studies related to eBC.

The association of mRNA expression of *ATM, BLM* and *WRN* with prognosis in eBC supports the development of personalized treatment strategies and therapeutic stratification. This could help identify patients who are at a higher risk of disease progression. High mRNA expression of *BLM*, along with low expression of *ATM* and *WRN*, may serve as prognostic biomarkers for pinpointing patients with worse survival outcomes. Incorporating these findings into molecular profiling panels for eBC could aid in personalizing standard treatment approaches and optimizing therapeutic outcomes. Further validation in larger cohorts and clinical trials is essential before these findings can be routinely implemented.

Understanding the role of *ATM, BLM and WRN* mRNA expression not only provides insights into potential prognostic biomarkers for eBC but also contributes to the development of genomic-based prognostic models. While these approaches necessitate further clinical validation, integrating DNA repair gene expression data into therapeutic decision-making holds promise for advancing precision oncology in eBC.

Future research should focus on validating these results in larger multicenter cohorts and exploring the underlying mechanisms to develop new genomic-based strategies for selecting eBC patients prior to treatment.

## 5 Conclusions

This study identifies high levels of *ATM* and *WRN* mRNA expression, along with low levels of *BLM* mRNA expression, as potential favorable prognostic markers for patients with eBC. Incorporating these biomarkers into clinical practice could enhance treatment and follow-up strategies for patients with eBC. Our analysis represents a significant step toward developing improved genomic-based prognostic algorithms.

## Supplementary Materials









## Data Availability

The main data supporting the findings of this study are available within the paper and its Supplementary Information. The datasets used and analyzed during the current study are available from the corresponding author on reasonable request.

## Supplementary Materials

The supplementary material is available online at https://www.techscience.com/doi/10.32604/or.2025.072222/s1.

## References

[1] Haibe-Kains B, Desmedt C, Loi S, Culhane AC, Bontempi G, Quackenbush J, et al. A three-gene model to robustly identify breast cancer molecular subtypes. J Natl Cancer Inst. 2012;104(4):311–25. doi:10.1093/jnci/djr545; 22262870 PMC3283537

[2] Heimes AS, Almstedt K, Krajnak S, Runkel A, Droste A, Schwab R, et al. Prognostic impact of LAG-3 mRNA expression in early breast cancer. Biomedicines. 2022;10(10):2656. doi:10.3390/biomedicines10102656; 36289918 PMC9599264

[3] Schmidt M, Böhm D, von Törne C, Steiner E, Puhl A, Pilch H, et al. The humoral immune system has a key prognostic impact in node-negative breast cancer. Cancer Res. 2008;68(13):5405–13. doi:10.1158/0008-5472.CAN-07-5206; 18593943

[4] Kurebayashi J, Yamamoto Y, Kurosumi M, Okubo S, Nomura T, Tanaka K, et al. Loss of BRCA1 expression may predict shorter time-to-progression in metastatic breast cancer patients treated with taxanes. Anticancer Res. 2006;26(1B):695–701; 16739340

[5] Shehaj I, Krajnak S, Almstedt K, Degirmenci Y, Herzog S, Lebrecht A, et al. BRCA1, BRCA2 and PALB2 mRNA expression as prognostic markers in patients with early breast cancer. Biomedicines. 2024;12(6):1361. doi:10.3390/biomedicines12061361; 38927568 PMC11202204

[6] Lee JH, Paull TT. ATM activation by DNA double-strand breaks through the Mre11-Rad50-Nbs1 complex. Science. 2005;308(5721):551–4. doi:10.1126/science.1108297; 15790808

[7] Stucci LS, Internò V, Tucci M, Perrone M, Mannavola F, Palmirotta R, et al. The ATM gene in breast cancer: its relevance in clinical practice. Genes. 2021;12(5):727. doi:10.3390/genes12050727; 34068084 PMC8152746

[8] Miser-Salihoglu E, Demokan S, Karanlik H, Karahalil B, Önder S, Cömert S, et al. Investigation of mRNA expression levels of Tip60 and related DNA repair genes in molecular subtypes of breast cancer. Clin Breast Cancer. 2023;23(2):125–34. doi:10.1016/j.clbc.2022.10.012; 36463002

[9] Ye C, Cai Q, Dai Q, Shu XO, Shin A, Gao YT, et al. Expression patterns of the ATM gene in mammary tissues and their associations with breast cancer survival. Cancer. 2007;109(9):1729–35. doi:10.1002/cncr.22592; 17366603

[10] Bueno RC, Canevari RA, Villacis RAR, Domingues MAC, Caldeira JRF, Rocha RM, et al. ATM down-regulation is associated with poor prognosis in sporadic breast carcinomas. Ann Oncol. 2014;25(1):69–75. doi:10.1093/annonc/mdt421; 24285016

[11] Tommiska J, Bartkova J, Heinonen M, Hautala L, Kilpivaara O, Eerola H, et al. The DNA damage signalling kinase ATM is aberrantly reduced or lost in BRCA1/BRCA2-deficient and ER/PR/ERBB2-triple-negative breast cancer. Oncogene. 2008;27(17):2501–6. doi:10.1038/sj.onc.1210885; 17982490

[12] Chen CF, Brill SJ. Multimerization domains are associated with apparent strand exchange activity in BLM and WRN DNA helicases. DNA Repair. 2014;22:137–46. doi:10.1016/j.dnarep.2014.07.015; 25198671 PMC4174979

[13] Zhu X, Chen H, Yang Y, Xu C, Zhou J, Zhou J, et al. Distinct prognosis of mRNA expression of the five RecQ DNA-helicase family members—RECQL, BLM, WRN, RECQL4, and RECQL5—in patients with breast cancer. Cancer Manag Res. 2018;10:6649–68. doi:10.2147/CMAR.S185769; 30584360 PMC6287649

[14] Opresko PL, Calvo JP, von Kobbe C. Role for the Werner syndrome protein in the promotion of tumor cell growth. Mech Ageing Dev. 2007;128(7–8):423–36. doi:10.1016/j.mad.2007.05.009; 17624410

[15] Rossi ML, Ghosh AK, Bohr VA. Roles of Werner syndrome protein in protection of genome integrity. DNA Repair. 2010;9(3):331–44. doi:10.1016/j.dnarep.2009.12.011; 20075015 PMC2827637

[16] Agrelo R, Cheng WH, Setien F, Ropero S, Espada J, Fraga MF, et al. Epigenetic inactivation of the premature aging Werner syndrome gene in human cancer. Proc Natl Acad Sci U S A. 2006;103(23):8822–7. doi:10.1073/pnas.0600645103; 16723399 PMC1466544

[17] Heimes AS, Härtner F, Almstedt K, Krajnak S, Lebrecht A, Battista MJ, et al. Prognostic significance of interferon-γ and its signaling pathway in early breast cancer depends on the molecular subtypes. Int J Mol Sci. 2020;21(19):7178. doi:10.3390/ijms21197178; 33003293 PMC7582264

[18] Győrffy B. Survival analysis across the entire transcriptome identifies biomarkers with the highest prognostic power in breast cancer. Comput Struct Biotechnol J. 2021;19:4101–9. doi:10.1016/j.csbj.2021.07.014; 34527184 PMC8339292

[19] Rondeau S, Vacher S, De Koning L, Briaux A, Schnitzler A, Chemlali W, et al. ATM has a major role in the double-strand break repair pathway dysregulation in sporadic breast carcinomas and is an independent prognostic marker at both mRNA and protein levels. Br J Cancer. 2015;112(6):1059–66. doi:10.1038/bjc.2015.60; 25742469 PMC4366900

[20] Savva C, De Souza K, Ali R, Rakha EA, Green AR, Madhusudan S. Clinicopathological significance of ataxia telangiectasia-mutated (ATM) kinase and ataxia telangiectasia-mutated and Rad3-related (ATR) kinase in MYC overexpressed breast cancers. Breast Cancer Res Treat. 2019;175(1):105–15. doi:10.1007/s10549-018-05113-8; 30746633 PMC6491658

[21] Dahlstrom JE, Rakha EA, Lakhani SR, Schnitt SJ. Current topics in breast pathology: expert perspectives. Pathology. 2017;49(2):109–10. doi:10.1016/j.pathol.2016.12.001; 28069256

[22] Yiu TC, Tu J, Cheung HH. An overview of RecQ helicases and related diseases. Aging. 2025;17(7):1881–907. doi:10.18632/aging.206291; 40728512 PMC12339026

[23] Li XL, Lu X, Parvathaneni S, Bilke S, Zhang H, Thangavel S, et al. Identification of RECQ1-regulated transcriptome uncovers a role of RECQ1 in regulation of cancer cell migration and invasion. Cell Cycle. 2014;13(15):2431–45. doi:10.4161/cc.29419; 25483193 PMC4128887

[24] Thakkar MK, Lee J, Meyer S, Chang VY. RecQ helicase somatic alterations in cancer. Front Mol Biosci. 2022;9:887758. doi:10.3389/fmolb.2022.887758; 35782872 PMC9240438

[25] Arora A, Abdel-Fatah TMA, Agarwal D, Doherty R, Moseley PM, Aleskandarany MA, et al. Transcriptomic and protein expression analysis reveals clinicopathological significance of bloom syndrome helicase (BLM) in breast cancer. Mol Cancer Ther. 2015;14(4):1057–65. doi:10.1158/1535-7163.MCT-14-0939; 25673821

[26] Kawabe T, Tsuyama N, Kitao S, Nishikawa K, Shimamoto A, Shiratori M, et al. Differential regulation of human RecQ family helicases in cell transformation and cell cycle. Oncogene. 2000;19(41):4764–72. doi:10.1038/sj.onc.1203841; 11032027

[27] Birkbak NJ, Li Y, Pathania S, Greene-Colozzi A, Dreze M, Bowman-Colin C, et al. Overexpression of BLM promotes DNA damage and increased sensitivity to platinum salts in triple-negative breast and serous ovarian cancers. Ann Oncol. 2018;29(4):903–9. doi:10.1093/annonc/mdy049; 29452344 PMC5913643

[28] Shamanna RA, Lu H, Croteau DL, Arora A, Agarwal D, Ball G, et al. Camptothecin targets WRN protein: mechanism and relevance in clinical breast cancer. Oncotarget. 2016;7(12):13269–84. doi:10.18632/oncotarget.7906; 26959889 PMC4924640

[29] Uechi Y, Fujikane R, Morita S, Tamaoki S, Hidaka M. Bloom syndrome DNA helicase mitigates mismatch repair-dependent apoptosis. Biochem Biophys Res Commun. 2024;723:150214. doi:10.1016/j.bbrc.2024.150214; 38850810

[30] Multani AS, Chang S. WRN at telomeres: implications for aging and cancer. J Cell Sci. 2007;120(5):713–21. doi:10.1242/jcs.03397; 17314245

[31] Orren DK, Machwe A. Response to replication stress and maintenance of genome stability by WRN, the Werner syndrome protein. Int J Mol Sci. 2024;25(15):8300. doi:10.3390/ijms25158300; 39125869 PMC11311767

[32] Futami K, Ishikawa Y, Goto M, Furuichi Y, Sugimoto M. Role of Werner syndrome gene product helicase in carcinogenesis and in resistance to genotoxins by cancer cells. Cancer Sci. 2008;99(5):843–8. doi:10.1111/j.1349-7006.2008.00778.x; 18312465 PMC11158842

[33] Kategaya L, Perumal SK, Hager JH, Belmont LD. Werner syndrome helicase is required for the survival of cancer cells with microsatellite instability. iScience. 2019;13:488–97. doi:10.1016/j.isci.2019.02.006; 30898619 PMC6441948

[34] Arai A, Chano T, Futami K, Furuichi Y, Ikebuchi K, Inui T, et al. RECQL1 and WRN proteins are potential therapeutic targets in head and neck squamous cell carcinoma. Cancer Res. 2011;71(13):4598–607. doi:10.1158/0008-5472.CAN-11-0320; 21571861

[35] Huang AV, Kong Y, Wang K, Brown ML, Mu D. Protein marker-dependent drug discovery targeting breast cancer stem cells. Int J Mol Sci. 2025;26(16):7935. doi:10.3390/ijms26167935; 40869256 PMC12386411

[36] Al-Hajj M, Wicha MS, Benito-Hernandez A, Morrison SJ, Clarke MF. Prospective identification of tumorigenic breast cancer cells. Proc Natl Acad Sci U S A. 2003;100(7):3983–8. doi:10.1073/pnas.0530291100; 12629218 PMC153034

[37] Battula VL, Shi Y, Evans KW, Wang RY, Spaeth EL, Jacamo RO, et al. Ganglioside GD2 identifies breast cancer stem cells and promotes tumorigenesis. J Clin Invest. 2012;122(6):2066–78. doi:10.1172/jci59735; 22585577 PMC3591166

[38] Ricardo S, Vieira AF, Gerhard R, Leitão D, Pinto R, Cameselle-Teijeiro JF, et al. Breast cancer stem cell markers CD44, CD24 and ALDH1: expression distribution within intrinsic molecular subtype. J Clin Pathol. 2011;64(11):937–46. doi:10.1136/jcp.2011.090456; 21680574

[39] Weigelt B, Bi R, Kumar R, Blecua P, Mandelker DL, Geyer FC, et al. The landscape of somatic genetic alterations in breast cancers from ATM germline mutation carriers. J Natl Cancer Inst. 2018;110(9):1030–4. doi:10.1093/jnci/djy028; 29506079 PMC6136925

[40] Goldgar DE, Healey S, Dowty JG, Da Silva L, Chen X, Spurdle AB, et al. Rare variants in the *ATM* gene and risk of breast cancer. Breast Cancer Res. 2011;13(4):1–9. doi:10.1186/bcr2919; 21787400 PMC3236337

[41] Hu C, Hart SN, Gnanaolivu R, Huang H, Lee KY, Na J, et al. A population-based study of genes previously implicated in breast cancer. N Engl J Med. 2021;384(5):440–51. doi:10.1056/nejmoa2005936; 33471974 PMC8127622

[42] Shiloh Y, Ziv Y. The ATM protein kinase: regulating the cellular response to genotoxic stress, and more. Nat Rev Mol Cell Biol. 2013;14(4):197–210. doi:10.1038/nrm3546; 23847781

[43] Sinha S, Ng V, Novaj A, Zhu Y, Yazaki S, Pei X, et al. The cold immunological landscape of ATM-deficient cancers. J Immunother Cancer. 2025;13(5):e010548. doi:10.1136/jitc-2024-010548; 40350205 PMC12067784

[44] Chenevix-Trench G. Dominant negative ATM mutations in breast cancer families. J Natl Cancer Inst. 2002;94(3):205–15. doi:10.1093/jnci/94.3.205; 11830610

[45] Llinares-Burguet I, Sanoguera-Miralles L, García-Álvarez A, Esteban-Sánchez A, Caloca MJ, de la Hoya M, et al. Unproductive alternative splicing of ATM exon 7: mapping of critical regulatory elements and identification of 34 spliceogenic variants. J Mol Med. 2025;103(11):1447–60. doi:10.1007/s00109-025-02595-0; 40974429 PMC12675606

[46] Narod SA. Which genes for hereditary breast cancer? N Engl J Med. 2021;384(5):471–3. doi:10.1056/nejme2035083; 33471975

[47] Cunniff C, Djavid AR, Carrubba S, Cohen B, Ellis NA, Levy CF, et al. Health supervision for people with Bloom syndrome. Am J Med Genet Part A. 2018;176(9):1872–81. doi:10.1002/ajmg.a.40374; 30055079

[48] Kluźniak W, Wokołorczyk D, Rusak B, Huzarski T, Kashyap A, Stempa K, et al. Inherited variants in BLM and the risk and clinical characteristics of breast cancer. Cancers. 2019;11(10):1548. doi:10.3390/cancers11101548; 31614901 PMC6826355

[49] Bonache S, Esteban I, Moles-Fernández A, Tenés A, Duran-Lozano L, Montalban G, et al. Multigene panel testing beyond BRCA1/2 in breast/ovarian cancer Spanish families and clinical actionability of findings. J Cancer Res Clin Oncol. 2018;144(12):2495–513. doi:10.1007/s00432-018-2763-9; 30306255 PMC11813382

[50] Tedaldi G, Tebaldi M, Zampiga V, Danesi R, Arcangeli V, Ravegnani M, et al. Multiple-gene panel analysis in a case series of 255 women with hereditary breast and ovarian cancer. Oncotarget. 2017;8(29):47064–75. doi:10.18632/oncotarget.16791; 28423363 PMC5564544

