# Supplementary figures and images for: Prognostic Significance of DNA Repair Gene mRNA Expression in Early-Stage Breast Cancer: Insights into Clinical Relevance
Source: Oncol Res. 2026 Feb 24;34(3):11. doi: 10.32604/or.2025.072222 (PMC12963681; doi:10.32604/or.2025.072222)

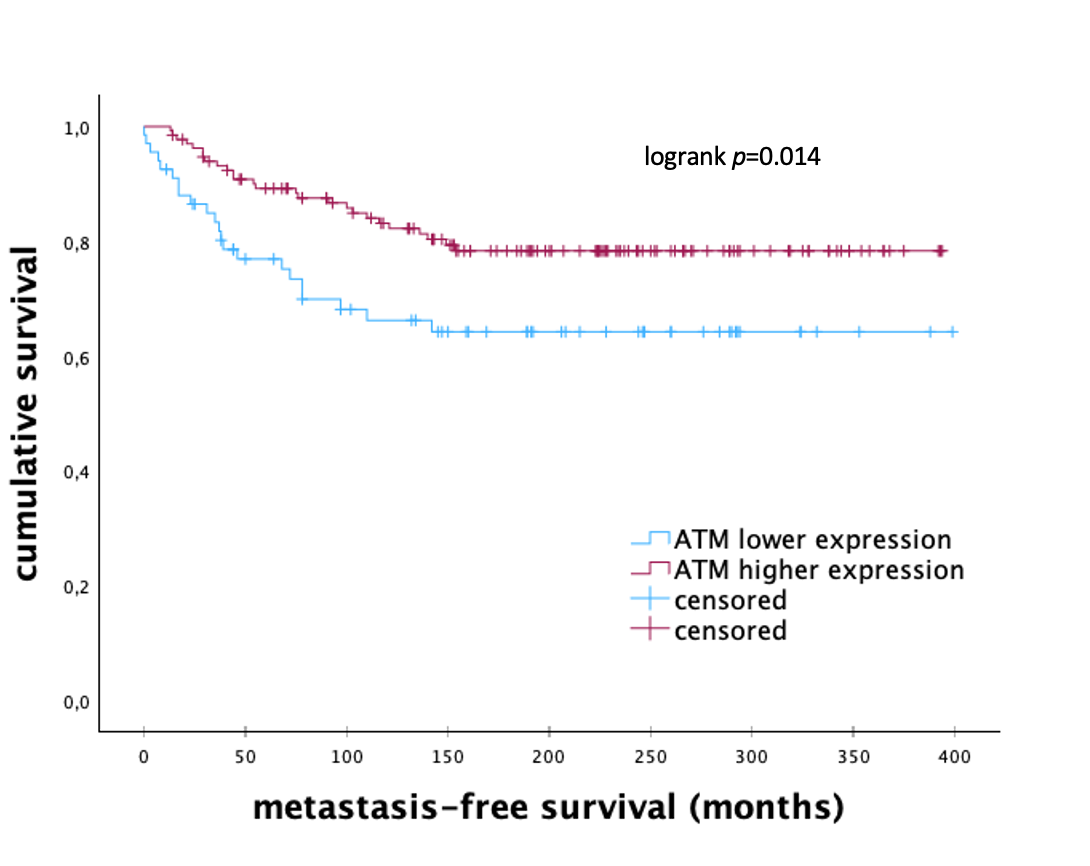

Supplement: Supplementary file 1 [file OncolRes-34-72222-s001.tiff]

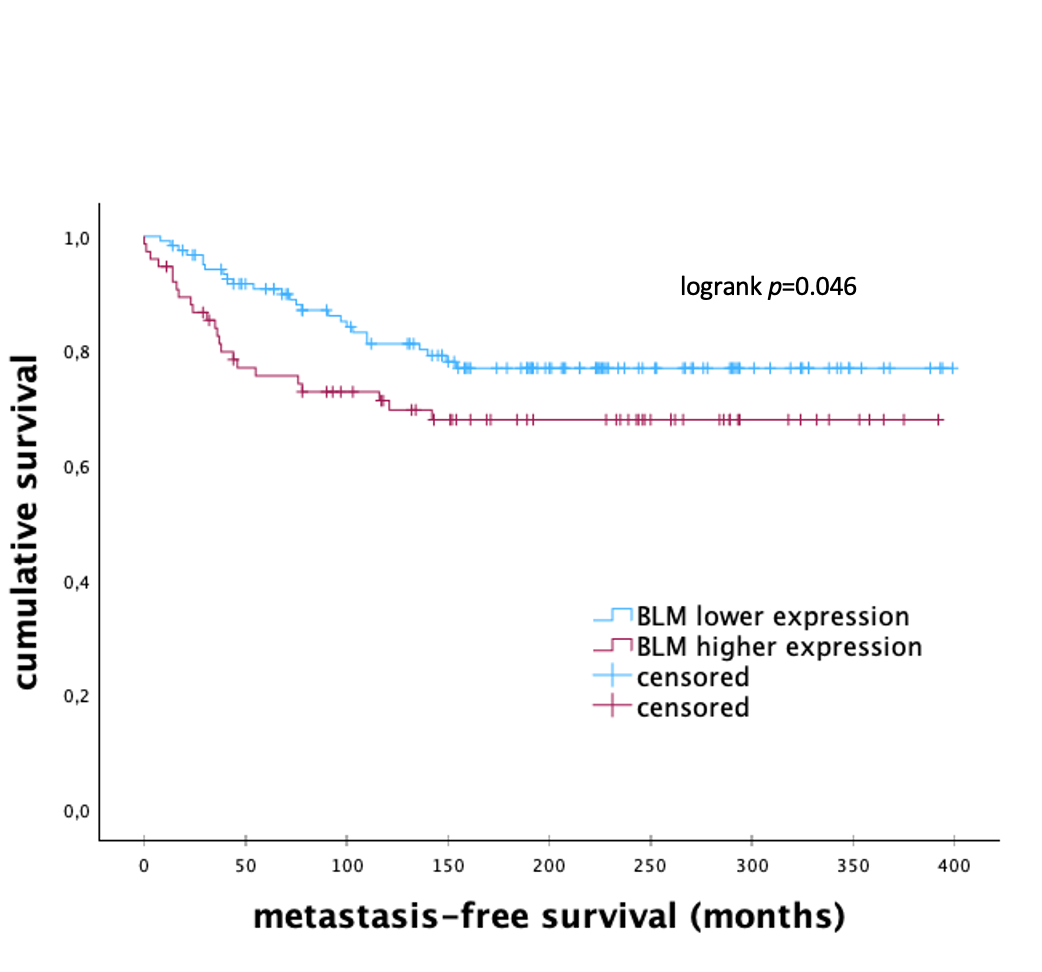

Supplement: Supplementary file 2 [file OncolRes-34-72222-s002.tiff]

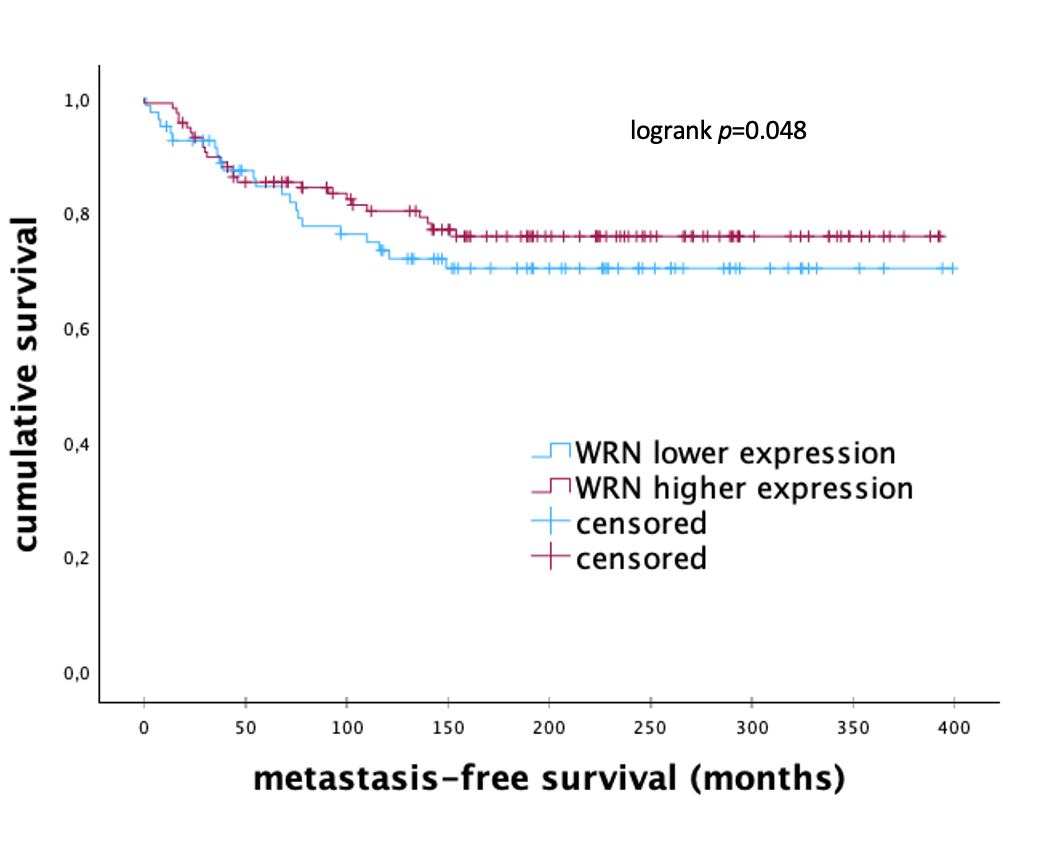

Supplement: Supplementary file 3 [file OncolRes-34-72222-s003.tiff]
