# Supplementary material for: Prognostic Significance of DNA Repair Gene mRNA Expression in Early-Stage Breast Cancer: Insights into Clinical Relevance
Source: Oncol Res. 2026 Feb 24;34(3):11. doi: 10.32604/or.2025.072222 (PMC12963681; doi:10.32604/or.2025.072222)
Supplement: Supplementary file 4 [file OncolRes-34-72222-s004.docx]

**Supplementary Material**


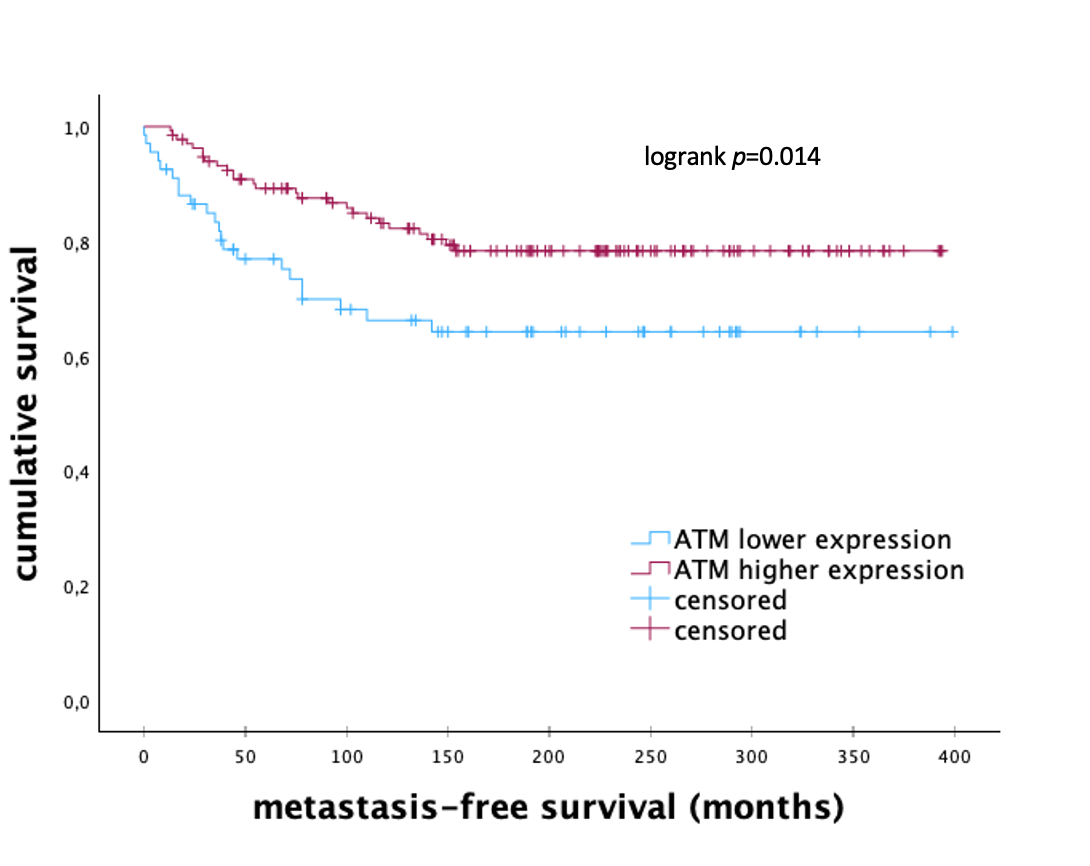


**Figure S1.** Kaplan–Meier analysis of metastasis-free survival in patients who did not receive adjuvant therapy (N0) with early breast cancer according to the levels of ATM mRNA expression, ATM, Ataxia Telangiectasia Mutated; *p*-value < 0.05 is considered significant.


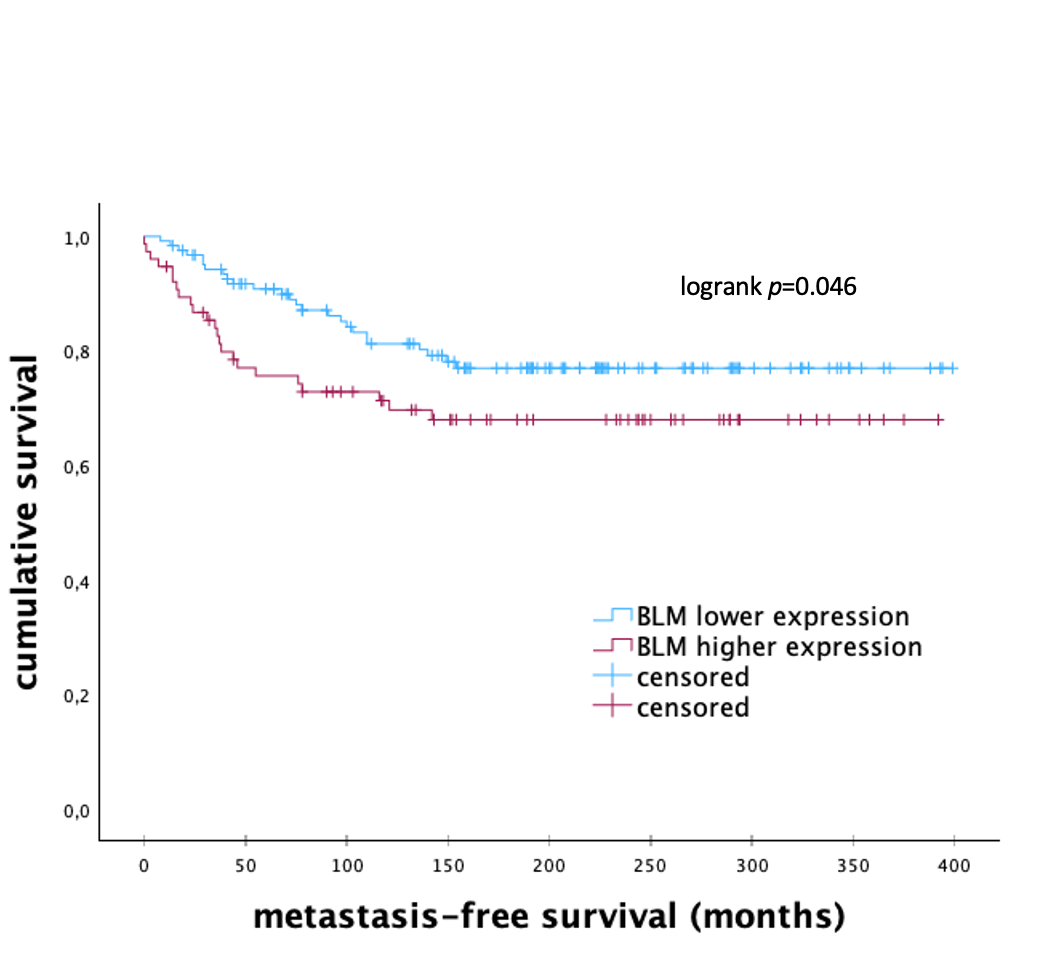


**Figure S2.** Kaplan–Meier analysis of metastasis-free survival in patients who did not receive adjuvant therapy (N0) with early breast cancer according to the levels of *BLM* mRNA expression. *BLM*, *Bloom helicase*; *p*-value < 0.05 is considered significant


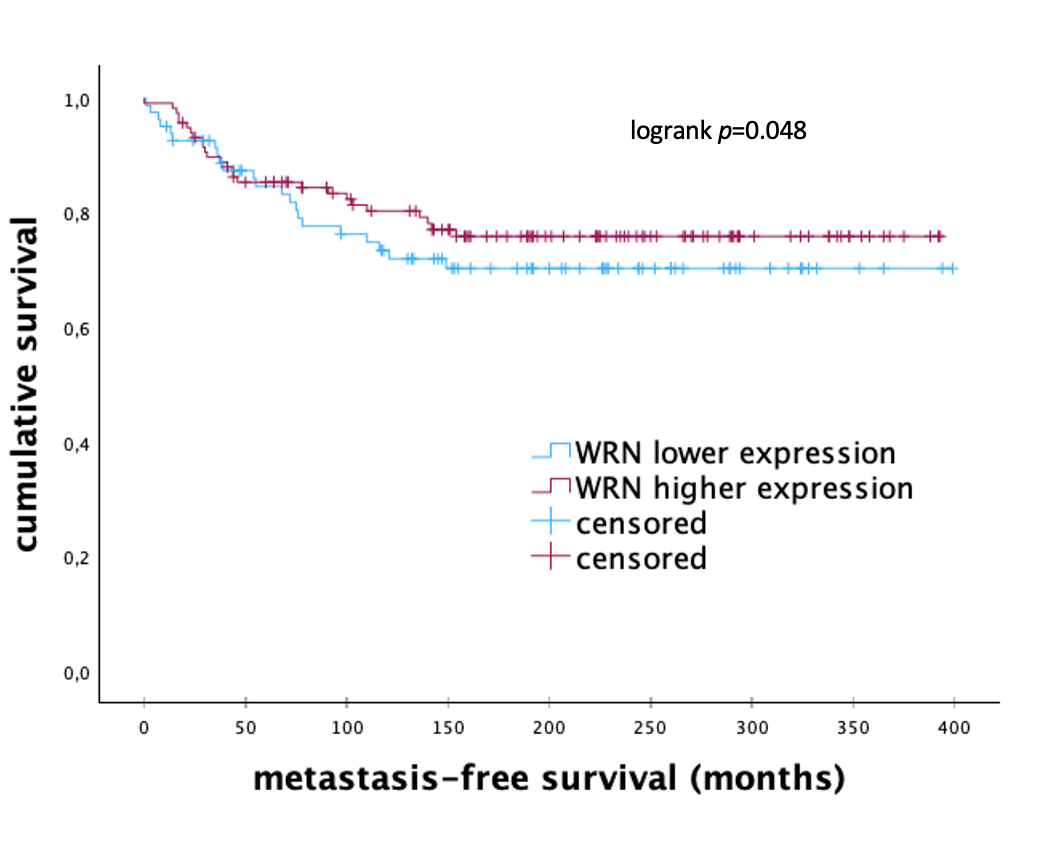


**Figure S3.** Kaplan–Meier analysis of metastasis-free survival in patients who did not receive adjuvant therapy (N0) with early breast cancer according to the levels of WRN mRNA expression. WRN, WRN RecQ Like Helicase; p-value < 0.05 is considered significant
